# Enabling access to molecular monitoring for chronic myeloid leukemia patients is cost effective in China

**DOI:** 10.1371/journal.pone.0259076

**Published:** 2021-10-25

**Authors:** Vikalp Kumar Maheshwari, Cassandra Slader, Nidhi Dani, Christina Gkitzia, Quan Yuan, Tengbin Xiong, Yu Liu, Ricardo Viana

**Affiliations:** 1 NBS CONEXTS–Value & Access, Novartis Healthcare Pvt. Ltd., Hyderabad, Telangana, India; 2 Novartis Pharma AG, Medical Affairs, Basel, Basel Stadt, Switzerland; 3 Novartis Pharma AG, Value & Access, Basel, Basel Stadt, Switzerland; 4 Novartis Oncology, Market Access Strategy, Beijing, China; 5 Novartis Oncology, Novartis Campus, Shanghai, China; 6 Novartis Oncology, Medical Affairs, Beijing, China; 7 Novartis Pharma AG, Global Value & Access, Oncology, Basel, Basel Stadt, Switzerland; Qatar University, QATAR

## Abstract

**Objective:**

To determine the cost effectiveness of molecular monitoring in patients with chronic myeloid leukemia in the chronic phase (CML-CP) compared to no molecular monitoring from a Chinese payer perspective.

**Methods:**

Analyses were conducted using a semi-Markov model with a 50-year time horizon. Population data from multicenter registry-based studies of Chinese patients with CML-CP informed the model. Transition probabilities were based on time-to-event data from the literature. Utility values were obtained from published studies and were assumed to be the same for patients with and without molecular monitoring. Costs were based on values commonly used in the Chinese healthcare system, including drug acquisition, drug administration, follow-up, treatment for disease progression, molecular monitoring, and terminal care costs, and were in the local currency (2020 Chinese Yuan RMB [¥]). Outcomes were total life-years (LYs) and quality-adjusted life years (QALYs), lifetime costs, and incremental cost-effectiveness ratio.

**Results:**

Molecular monitoring was dominant to no molecular monitoring, with increased LYs (1.52) and QALYs (1.90) and costs savings (¥93,840) over a lifetime compared to no monitoring in discounted analyses. The opportunity of patients that receive molecular monitoring to discontinue treatment during treatment-free remission, an opportunity not afforded to those without molecular monitoring, was the principle driver of this result. Results were similar across multiple clinical scenarios. Particularly, molecular monitoring remained dominant even if the proportion of patients achieving deep molecular response (DMR) was reduced by 10%-30%, or the proportion of patients maintaining DMR for 1 year was reduced by 10%-30% or increased by 10%. Cost savings in these scenarios ranged from ¥62,230 to ¥103,964.

**Conclusions:**

Overall, this analysis demonstrates that adherence to guideline recommendations of regular molecular monitoring of patients with CML-CP treated with TKIs provides significant clinical benefit that leads to substantial cost savings compared to no molecular monitoring from the perspective of a Chinese payer. In a time where healthcare systems have limited resources to allocate to optimal patient care, investment in molecular monitoring is an ideal choice for improving patient benefits at a reduced cost.

## Introduction

Chronic myeloid leukemia (CML), a myeloproliferative blood cancer, has an incidence of 0.39–0.55 cases per 100,000 adults in China [1]. The disease is characterized by a reciprocal translocation between chromosomes 9 and 22, giving rise to the Philadelphia chromosome encoding the *BCR-ABL1* gene [2,3]. Expression of the resulting BCR-ABL1 oncoprotein, with constitutive tyrosine kinase activity, leads to chronic dysregulation of key proliferation, apoptotic and cell adhesion pathways [4–7].

Patients typically present in the chronic phase of CML (CML-CP) before progressing to the more severe accelerated phase (AP) or blast crisis phase (BC) [8]. The standard of care for CML-CP patients in China is treatment with tyrosine kinase inhibitors (TKIs) such as imatinib, nilotinib, dasatinib and flumatinib. These have been shown to significantly reduce CML related mortality [9]. Notably, 5-year disease-specific survival improved from 47.3% to 80.8% after the introduction of TKIs [9]. Similar survival rates are reported in studies that included the Chinese population, with 92% of patients with CML-CP surviving beyond 5 years [10]. With greater overall survival, the cumulative costs associated with the long-term treatment of patients is a growing burden on the health care system. In further support of this notion, accumulating evidence demonstrates that patients with CML-CP that achieve a complete cytogenetic response have a similar overall survival as that of the general population [11]. It is now well-established that patients on TKI therapy can go on to achieve a deep molecular response (DMR), typically defined as *BCR-ABL1* transcript levels of ≤0.01% (MR4) or <0.0032% (MR4.5) on the International Scale [12,13]. Evidence from several studies suggest that approximately 50% of patients who achieve a sustained stable DMR may safely discontinue TKI treatment without relapse, with routine molecular monitoring [14–19]. As such, treatment-free remission (TFR) is an important treatment goal among CML-CP patients [13]. Current guidelines recommend discontinuation of TKI treatment in patients who have been treated with approved TKI for at least 5 years, achieved sustained DMR (MR4) after at least 2 years of treatment, and have no history of accelerated or blast phase CML. Although current guidelines do not differentiate between TKIs that are currently available, TFR is only included in the approved indication of nilotinib. Additional guideline criteria for TFR include regular molecular monitoring every month during the first 6 months of TFR, every 8 weeks for months 6–12, and every 12 weeks thereafter [13,20,21]. During TFR, the clinical course for patients that fail to maintain a DMR is to restart TKI treatment immediately [22].

Routine molecular monitoring using quantitative polymerase chain reaction (qPCR) is recommended to assess disease progression and response to TKI treatment [13,21,23–25]. Despite this, several studies have revealed that as few as 50% of patients receiving TKI treatment undergo molecular monitoring during the first years of treatment [26–29]. Reduced monitoring of patients may lead to increased disease progression to the AP and BC phases, which are associated with substantial burden and high costs [30]. Economic analyses have reported reductions in healthcare resource utilization and cost savings with adherence to guideline recommendations for molecular monitoring [31–34]. However, the cost effectiveness of molecular monitoring in the context of TKI treatment of CML-CP is currently unknown. In addition, based on the treatment guideline criteria, molecular monitoring continues to play a pivotal role during TFR; ensuring that patients remain free of molecular relapse. To date, progression to AP/BC directly from TFR has not been reported in clinical trials although there have been isolated case reports in the literature [35]. The objective of this study was to determine the cost effectiveness of molecular monitoring in patients with CML-CP who are receiving standard CML treatment to no molecular monitoring from a Chinese payer perspective.

## Methods

### Model overview and design

A semi-Markov model was developed to compare benefits and costs associated with molecular monitoring to that associated with no molecular monitoring in CML-CP patients from a Chinese payer perspective. Analyses were conducted for a lifetime (ie, 50-year) time horizon. Cycle length was 1-year and half-cycle correction was applied. Benefits were measured in total life-years (LYs) and quality-adjusted life years (QALYs), and lifetime costs were calculated in the local currency Chinese Yuan (RMB ¥). The incremental cost-effectiveness ratio (ICER) was estimated by dividing the difference in lifetime costs between the two groups by the difference in benefits (ie, LYs or QALYs).

**Fig 1:**
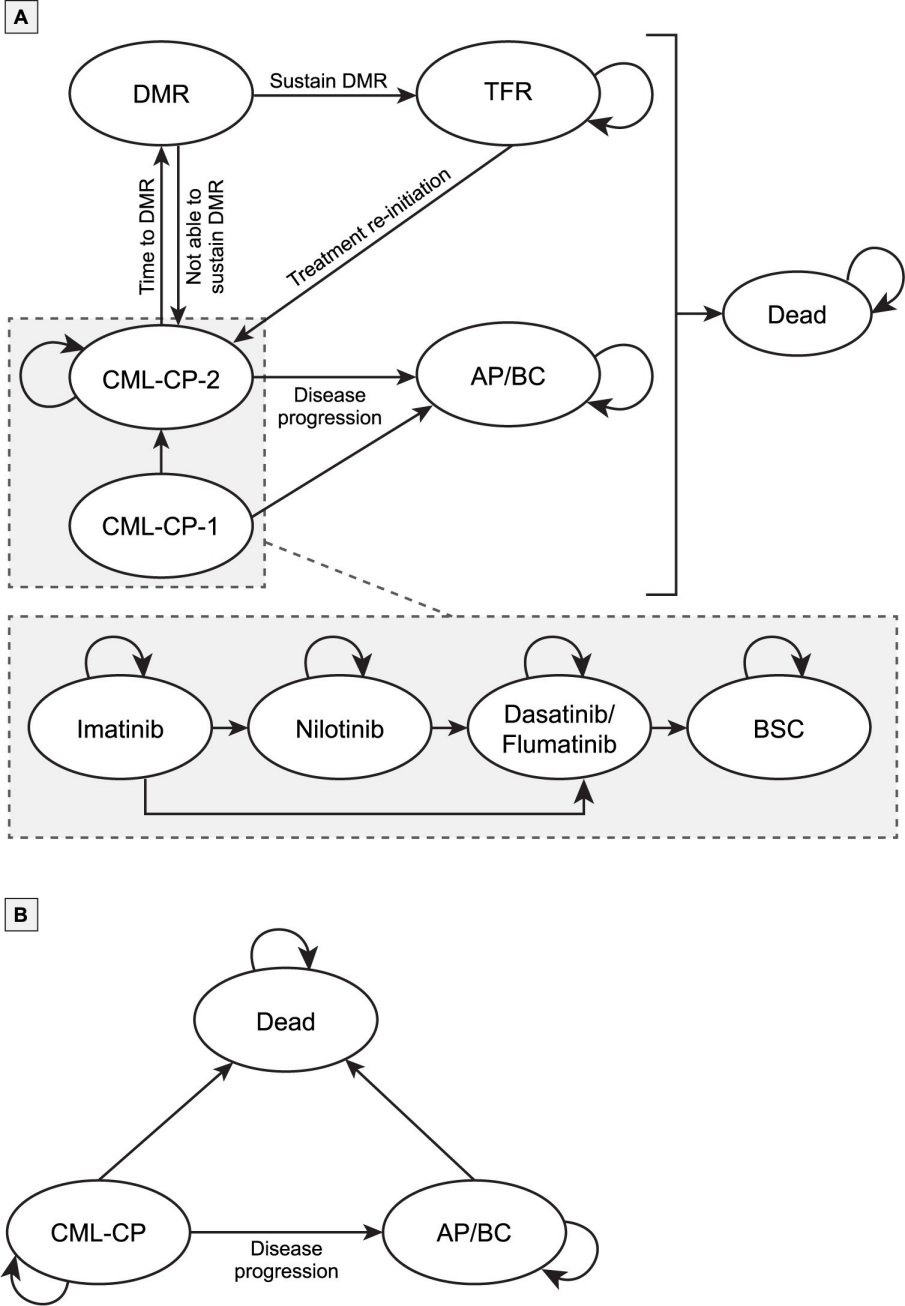
Structure of the Markov model. Abbreviations: AP, Accelerated Phase; BC, Blast Crisis; CML-CP, Chronic Myeloid Leukemia; DMR, Deep Molecular Response; TFR, Treatment Free Remission.

The model structure was developed based on whether patients would or would not be monitored using BCR-ABL quantitative PCR [subsequently referred to as molecular monitoring] (Fig 1). All patients started treatment with imatinib, nilotinib, or dasatinib. Patients that achieved DMR were assumed to remain on the same therapy due to high conditional probability for transformation free survival and overall survival after 12 months of treatment with TKIs [36]. Bosutinib and ponatinib are not currently approved in China and were therefore excluded from the model. Flumatinib, a locally developed and approved second generation TKI is included. In the absence of time to treatment discontinuation (TTD) and progression-free survival (PFS) curves for flumatinib, flumatinib was assumed to have similar efficacy as dasatinib, therefore, patients on flumatinib were grouped under dasatinib. On treatment discontinuation, patients moved to the next TKI (eg, patients who discontinued imatinib were moved to either nilotinib or dasatinib; patients who discontinued nilotinib were moved to dasatinib; patients who discontinued dasatinib were moved to best supportive care [BSC] [imatinib + interferon (IFN)]). Because molecular monitoring allows physicians to identify patients who achieve DMR, patients who were monitored either transitioned to the DMR or progressed to the AP/BC health state (Fig 1A). Patients unable to sustain DMR transitioned back to the CML-CP health state, whereas patients with sustained DMR transitioned to the TFR health state. The model assumed that only patients who were treated with TKIs and not BSC were able to achieve DMR and attempt TFR. Patients who achieved the TFR health state either remained in that state until death or until the loss of MMR at which point they were transitioned back to the CML-CP health state. Patients who progressed to the AP/BC health state remained in that state until death.

Without regular monitoring, physicians cannot identify if the patient has achieved DMR and hence are not eligible for treatment discontinuation/TFR [37]. As such, CML-CP patients who did not receive molecular monitoring remained in the CML-CP health state, transitioned to the AP/BC health state, or transitioned to death (Fig 1B).

### Patient population and model inputs

All patients included in our model were newly diagnosed with CML-CP. Population data used to inform the model were derived from multicenter registry-based studies of Chinese patients with CML-CP [1,38,39] (Table 1). The mean age of patients included in the study was 41, and 62% were assumed to be males [39]. Differences in the mean age of patients diagnosed with CML-CP between this study and others conducted in western nations are most likely attributable to regional differences [39]. For the base case analysis, 75% of CML-CP patients received imatinib, 13% received nilotinib, and 12% received dasatinib/flumatinib as first-line treatments based on local market research data [40]. The percentage of patients achieving DMR was based on the time to MR4.5 curves for imatinib, nilotinib and dasatinib. The probability of maintaining DMR was 57% with imatinib, 77% with nilotinib, and 72% with dasatinib [41–43]. It was assumed that 50% of patients received imatinib and 50% dasatinib in the AP/BC health state. Death of patients in the CML-CP, DMR, and TFR health states was based on general/all-cause mortality data from the Chinese population [44].

**Table 1:** Clinical inputs for base case analysis.

Parameter	Base Case Value	Source
% male	62%	[39]
Discount rate (%) Costs Benefits	3% 3%	[45,46]
Patient distribution on 1^st^ line TKIs* Imatinib Nilotinib Dasatinib (Flumatinib)**	75% 13% 12%	[40]
Proportion of patients achieving DMR Imatinib Nilotinib Dasatinib (Flumatinib)**	DMR curves	Assumption
Probability of maintaining DMR for 1 year Imatinib Nilotinib Dasatinib (Flumatinib)**	57% 77% 72%	[41] [43] [42]
Patient distribution in AP/BC health states Imatinib Dasatinib (Flumatinib)**	50% 50%	Assumption based on clinician input
Transition probabilities from Imatinib to: Nilotinib Dasatinib (Flumatinib)**	50% 50%	Assumption based on clinician input
Transition probabilities from Nilotinib to: Dasatinib (Flumatinib)** BSC	100% 0%	Assumption (last line treatment option)
Transition probabilities from Dasatinib to: BSC	100%	Assumption
Utility values for patients with monitoring CML-CP DMR TFR AP/BC	0.854 0.940 1.000 0.595	[47] [48] [49] [47]
Utility values for patients without monitoring CML-CP AP/BC	0.854 0.595	Assumption: same as that of with monitoring
Frequency of molecular testing CML-CP DMR TFR (1^st^ year) TFR (2^nd^ + years)	2.87 per year 2.5 per year 9 per year 2 per year	Based on input from key opinion leaders
Nurse-led visit CML-CP DMR TFR AP/BC	1.52 per year 1.16 per year 1.16 per year 2.04 per year	Single Technology Appraisal-Ponatinib for treating chronic myeloid leukaemia and acute lymphoblastic leukaemia-Committee Papers (2017)—NICE Technology Appraisal Guidance [TA451]
Haematologist-led visit CML-CP DMR TFR AP/BC	6.88 per year 3.72 per year 3.72 per year 14.52 per year	
X-rays/radiography CML-CP DMR TFR AP/BC	0.00 per year 0.00 per year 0.00 per year 3.96 per year	
CT scans CML-CP DMR TFR AP/BC	0.00 per year 0.00 per year 0.00 per year 0.96 per year	
Full blood count CML-CP DMR TFR AP/BC	7.88 per year 4.52 per year 4.52 per year 17.52 per year	
Cytogenetic analysis CML-CP DMR TFR AP/BC	2.96 per year 2.32 per year 2.32 per year 3.60 per year	
Bone marrow aspiration (with biopsy) CML-CP DMR TFR AP/BC	1.20 per year 0.12 per year 0.12 per year 3.60 per year	
FISH test CML-CP DMR TFR AP/BC	2.24 per year 0.88 per year 0.88 per year 0.52 per year	
Blood film exam CML-CP DMR TFR AP/BC	4.36 per year 2.00 per year 2.00 per year 8.76 per year	
Blood chemistry CML-CP DMR TFR AP/BC	7.52 per year 4.52 per year 4.52 per year 12.60 per year	
Blood transfusion CML-CP DMR TFR AP/BC	0.04 per year 0.04 per year 0.04 per year 7.92 per year	
Platelet transfusion CML-CP DMR TFR AP/BC	0.00 per year 0.00 per year 0.00 per year 1.20 per year	
Cytochemistry analysis CML-CP DMR TFR AP/BC	0.20 per year 0.00 per year 0.00 per year 0.48 per year	
Hospital stay (days) AP/BC	36 per year	[50]

Abbreviations: AP, Accelerated Phase; BC, Blast Crisis; BSC, Imatinib + interferon alpha; CML-CP, Chronic Myeloid Leukemia; DMR, Deep Molecular Response; TFR, Treatment Free Remission; TKIs, tyrosine kinase inhibitors; TTD, time to discontinuation.

*The impact of mutations such as T315I was not considered among patients on first-line TKIs.

**The reference belonging to this row of the table refers to dasatinib. Due to limited data availability at the time of analyses, flumatinib was considered to have similar efficacy to dasatinib and therefore grouped with dasatinib.

Probabilities for transitioning from imatinib to nilotinib and dasatinib were 50% each, and those for transitioning from nilotinib to dasatinib and from dasatinib to BSC were 100% (Table 1). Transition probabilities between treatments and transition to death from the AP/BC health state were based on time-to-event data from the literature. Time-to-event data to inform transition probabilities for TTD, PFS, and OS were identified from publicly available systematic literature reviews, economic analyses, and pivotal clinical trials. Kaplan-Meier curves for TTD by treatment [51,52], PFS by treatment [15,52–54] and OS for AP/BC health state [55] were digitized using Plot Digitizer 2.6.6 software and curves were fit to the data using exponential, gamma, generalized gamma, log-normal, log-logistic, Gompertz, Weibull, RCS Weibull, and RCS log-logistic equations. A PFS curve for imatinib + interferon was not available for previously treated patients; therefore, a PFS curve for hydroxyurea + interferon was used. Curve fit was assessed visually and by the Akaike information criterion (AIC), where lower values indicated better fit (S1 Fig). The model assumed no change in TTD or PFS curves due to monitoring (i.e., the same curves were considered for patients with and without molecular monitoring).

Utility values were obtained from published studies and importantly were assumed to be the same for patients with and without molecular monitoring [47–49] (Table 1). Frequency of molecular testing was based on input from clinical experts. Resource utilization frequencies were obtained from technology appraisals from the National Institute for Health and Care Excellence (Table 1). Resource utilization considered in the model included nurse-led visit, haematologist-led visit, X-rays/radiography, CT scans, full blood count, cytogenetic analysis, bone marrow aspiration (with biopsy), FISH test, blood film exam, blood chemistry, blood transfusion, platelet transfusion, cytochemistry analysis, and hospital stay.

Cost inputs are summarized in (Table 2) and were based on values commonly used in the Chinese healthcare system [56]. Healthcare costs considered in the analysis included the following: drug acquisition costs, drug administration costs, follow-up costs, costs of progressed treatment, costs of molecular monitoring, and terminal care costs. Regarding the drug cost of dasatinib/flumatinib, the cost of dasatinib was used in the model rather than flumatinib, as the dasatinib cost was higher. Best supportive care costs considered the costs of imatinib and interferon. The cost of monitoring was based on values commonly used in the Chinese healthcare system [57]. Cost and disutility associated with adverse events (AEs) were not considered in the analysis.

**Table 2:** Cost inputs for base case analysis.

Parameter	Base Case Value	References
Drug cost (list price) Imatinib Nilotinib Dasatinib (flumatinib)* Interferon alpha	¥586.00 ¥11,364.00 ¥7,500.00 ¥912.88	[58] [58] [58] [56]
Drug administration cost Interferon alpha	¥1,095.75	[56]
Total follow-up resource use costs by health state CML-CP DMR TFR AP/BC	¥6,946.80 ¥3,926.80 ¥3926.80 ¥14,574.80	Calculated based on resource utilization
Molecular monitoring cost	¥250.00	[56]
Individual resource use follow-up unit costs Nurse-led visit Haematologist-led visit X-rays/radiography CT scans Full blood count Cytogenetic analysis Bone marrow aspiration (with biopsy) FISH test Blood film exam Blood chemistry Blood transfusion Platelet transfusion Cytochemistry analysis	¥30.00 ¥40.00 ¥70.00 ¥170.00 ¥20.00 ¥490.00 ¥100.00 ¥800.00 ¥100.00 ¥350.00 ¥450.00 ¥1,420.00 ¥100.00	[56]
Hospital stay (days)	¥300.00	[56]

Abbreviations: AP, Accelerated Phase; BC, Blast Crisis; CML-CP, Chronic Myeloid Leukemia; CT, computed tomography; DMR, Deep Molecular Response; TFR, Treatment Free Remission.

*The model considered the cost of dasatinib instead of flumatinib, as it was costlier.

### Scenario analyses

Multiple analyses were conducted to evaluate the impact of plausible clinical scenarios on model results (S1 Table). Scenarios evaluated changes in the proportion of male patients, discount rates, patient distribution on first-line TKIs, proportion of patients achieving DMR, proportion of patients maintaining DMR, transition probabilities from imatinib to nilotinib and from nilotinib to dasatinib, health-state utility values, frequency of molecular monitoring, and drug costs.

### Sensitivity analyses

One-way deterministic sensitivity analyses were performed to evaluate uncertainty of key parameters and to test model robustness. Key model input parameters were varied individually by ±25% of the base case value. Parameters examined in the sensitivity analyses included the utility values for patients in the CML-CP and AP/BC health states (with and without molecular monitoring), utility values for the TFR and DMR health state (with monitoring), probability of maintaining DMR for a year with dasatanib and nilotinib, mean age, and discount rates for benefits.

## Results

### Base case results

Undiscounted and discounted results showed increased LYs and QALYs and reduced costs with implementation of molecular monitoring compared to no molecular monitoring (Table 3). In the undiscounted analysis, implementation of molecular monitoring increased LYs and QALYs compared to no monitoring, with incremental LYs of 3.37 and incremental QALYs of 3.94. Implementation of molecular monitoring also reduced total cost compared to no monitoring, resulting in savings of ¥132,787 over a lifetime horizon. In the discounted analysis, molecular monitoring increased LYs and QALYs and reduced total costs compared to no monitoring, with incremental LYs of 1.52, incremental QALYs of 1.90, and savings of ¥93,840. Treatment of CML-CP patients in combination with monitoring was dominant compared to no monitoring, both in terms of cost per LYs gained and cost per QALYs gained.

**Table 3:** Base case analysis results.

	Undiscounted			Discounted			ICER	
	LY	QALYs	Cost	LY	QALYs	Cost	Cost per LY	Cost per QALY
With molecular monitoring	22.06	19.22	¥917,869	15.15	13.09	¥663,250		
Without molecular monitoring	18.69	15.28	¥1,050,656	13.63	11.19	¥757,090		
**Δ**	**3.37**	**3.94**	**-¥132,787**	**1.52**	**1.90**	**-¥93,840**	**Dominant**	**Dominant**

Abbreviations: **Δ**, change; ICER, incremental cost-effectiveness ratio; LY, life year; QALY, quality-adjusted life year.

Incremental LYs, incremental QALYs, and incremental costs varied by health state and follow-up treatment in undiscounted and discounted analyses (Table 4). In the CML-CP health state, molecular monitoring resulted in lower LYs (undiscounted Δ: -2.74; discounted Δ: -1.90), QALYs (undiscounted Δ: -2.34; discounted Δ: -1.62), and costs (undiscounted Δ: -¥167,693; discounted Δ: -¥119,844) than no molecular monitoring.

**Table 4:** Base case analysis results by health state.

	LY			QALY			Cost		
	With Molecular Monitoring	Without Molecular Monitoring	Δ LY	With Molecular Monitoring	Without Molecular Monitoring	Δ QALY	With Molecular Monitoring	Without Molecular Monitoring	Δ Cost
**Undiscounted**									
**CML-CP**	13.32	16.06	-2.74	11.37	13.71	-2.34	¥686,186	¥853,879	-¥167,693
**DMR**	0.63	0.000	0.63	0.59	0.00	0.59	¥31,429	¥0	¥31,429
**TFR**	6.00	0.000	6.00	6.00	0.00	6.00	¥0	¥0	¥0
**AP/BC**	2.12	2.64	-0.52	1.26	1.57	-0.31	¥30,212	¥37,628	-¥7,417
**Terminal care cost**	-	-	-	-	-	-	¥7,367	¥9,176	-¥1,809
**Follow-up cost**	-	-	-	-	-	-	¥149,384	¥149,973	-¥589
**Molecular monitoring cost**	-	-	-	-	-	-	¥13,292	¥0	¥13,292
**Total**	**22.06**	**18.69**	**3.37**	**19.22**	**15.28**	**3.94**	**¥917,869**	**¥1,050,656**	**-¥132,787**
**Discounted**									
**CML-CP**	10.00	11.90	-1.90	8.54	10.16	-1.62	¥499,085	¥618,929	-¥119,844
**DMR**	0.52	0.00	0.52	0.49	0.00	0.49	¥25,488	¥0	¥25,488
**TFR**	3.23	0.00	3.23	3.23	0.00	3.23	¥0	¥0	¥0
**AP/BC**	1.40	1.73	-0.32	0.84	1.03	-0.19	¥20,014	¥24,618	-¥4,604
**Terminal care cost**	-	-	-	-	-	-	¥4,647	¥5,716	-¥1,069
**Follow-up cost**	-	-	-	-	-	-	¥104,617	¥107,827	-¥3,209
**Molecular monitoring cost**	-	-	-	-	-	-	¥9,399	¥0	¥9,399
**Total**	**15.15**	**13.63**	**1.52**	**13.09**	**11.19**	**1.90**	**¥663,250**	**¥757,090**	**-¥93,840**

Abbreviations: **Δ**, change; AP, Accelerated Phase; BC, Blast Crisis; CML-CP, Chronic Myeloid Leukemia; DMR, Deep Molecular Response; LY, life years; QALY, quality-adjusted life year; TFR, Treatment Free Remission.

In the DMR health state, molecular monitoring had greater LYs (undiscounted Δ: 0.63; discounted Δ: 0.52), QALYs (undiscounted Δ: 0.59; discounted Δ: 0.49), and costs (undiscounted Δ: ¥31,429; discounted Δ: ¥25,488) than no molecular monitoring. Since molecular response status was not available for those patients who are not monitored with molecular monitoring, no patients moved to the DMR or TFR health states (ie, patients remained in the CML-CP health state). Hence no costs were incurred in the DMR or TFR health states. Likewise, in the TFR health state, molecular monitoring had greater LYs (undiscounted Δ: 6.00; discounted Δ: 3.23) and QALYs (undiscounted Δ: 6.00; discounted Δ: 3.23) than no molecular monitoring. Although no drug costs were associated with the TFR state, TFR was associated with monitoring and follow-up costs. In the AP/BC health state, molecular monitoring resulted lower LYs (undiscounted Δ: -0.52; discounted Δ: -0.32), QALYs (undiscounted Δ: -0.31; discounted Δ: -0.19), and costs (undiscounted Δ: -¥7,417; discounted Δ: -¥4,604) than no molecular monitoring. Terminal care costs were lower with molecular monitoring than without (undiscounted Δ: -¥1,809; discounted Δ: -¥1,069) and follow-up costs were slightly lower with molecular monitoring that without (undiscounted Δ: -¥589; discounted Δ: -¥3,209). Costs for molecular monitoring were ¥13,292 (undiscounted) and ¥9,399 (discounted).

### Scenario analysis results

Analyses investigating the impact of different clinical scenarios showed that molecular monitoring was the dominant strategy when compared to no molecular monitoring, with greater LYs and QALYs gained and lower costs across all scenarios (Table 5). Notably, reduction in the proportion of patients achieving the DMR health state by 10%, 20%, or 30% resulted in molecular monitoring being dominant over no molecular monitoring, with greater LYs (10% reduction: 1.47; 20% reduction: 1.43; 30% reduction: 1.37) and QALYs (10% reduction: 1.84; 20% reduction: 1.79; 30% reduction: 1.72), and lower costs (10% reduction: -¥91,208; 20% reduction: -¥88,440; 30% reduction -¥85,399). Similarly, variation in proportion of patients maintaining the DMR health state for 1 year, either by a reduction of 10%, 20%, or 30% or an increase of 10%, resulted in molecular monitoring being dominant to no molecular monitoring, with greater LYs (10% reduction: 1.41; 20% reduction: 1.30; 30% reduction: 1.19; 10% increase: 1.62) and QALYs (10% reduction: 1.76; 20% reduction: 1.61; 30% reduction: 1.47; 10% increase: 2.03), and lower costs (10% reduction: -¥83,511; 20% reduction: -¥72,975; 30% reduction: -¥62,230; 10% increase: -¥103,964).

**Table 5:** Scenario analysis results.

	With Molecular Monitoring			Without Molecular Monitoring			Incremental			
Description	Cost	LY	QALY	Cost	LY	QALY	Cost	LY	QALY	Cost per QALY
**Base Case**	**¥663,250**	**15.15**	**13.09**	**¥757,090**	**13.63**	**11.19**	**-¥93,840**	**1.52**	**1.90**	**Dominant**
All males	¥656,394	14.94	12.90	¥749,433	13.49	11.08	-¥93,040	1.45	1.82	Dominant
No discounting	¥917,869	22.06	19.22	¥1,050,656	18.69	15.28	-¥132,787	3.37	3.94	Dominant
5% discount	¥554,473	12.43	10.70	¥630,327	11.48	9.45	-¥75,853	0.95	1.24	Dominant
Imatinib -30%, Nilotinib -40% and rest dasatinib	¥767,866	14.64	12.68	¥872,099	12.93	10.57	-¥104,233	1.71	2.11	Dominant
Movement from imatinib to nilotinib -40%	¥628,171	14.95	12.89	¥711,816	13.44	11.02	-¥83,644	1.51	1.87	Dominant
Movement from imatinib to nilotinib -50%	¥663,250	15.15	13.09	¥757,090	13.63	11.19	-¥93,840	1.52	1.90	Dominant
Movement from nilotinib to dasatinib -10%	¥789,168	14.77	12.70	¥918,546	13.34	10.93	-¥129,379	1.43	1.77	Dominant
Movement from imatinib to nilotinib -30%	¥761,186	14.86	12.79	¥882,667	13.40	10.99	-¥121,481	1.45	1.80	Dominant
10% reduction in achieving DMR	¥664,995	15.10	13.03	¥756,204	13.63	11.19	-¥91,208	1.47	1.84	Dominant
20% reduction in achieving DMR	¥666,877	15.05	12.98	¥755,318	13.63	11.19	-¥88,440	1.43	1.79	Dominant
30% reduction in achieving DMR	¥669,032	15.00	12.91	¥754,431	13.63	11.19	-¥85,399	1.37	1.72	Dominant
10% less DMR Maintenance	¥673,579	15.04	12.95	¥757,090	13.63	11.19	-¥83,511	1.41	1.76	Dominant
20% less DMR maintenance	¥684,115	14.93	12.80	¥757,090	13.63	11.19	-¥72,975	1.30	1.61	Dominant
30% less DMR maintenance	¥694,861	14.82	12.66	¥757,090	13.63	11.19	-¥62,230	1.19	1.47	Dominant
10% higher DMR maintenance	¥653,126	15.25	13.22	¥757,090	13.63	11.19	-¥103,964	1.62	2.03	Dominant
10% less utility	¥663,250	15.15	11.78	¥757,090	13.63	10.07	-¥93,840	1.52	1.71	Dominant
Frequency of monitoring based on ELN guidelines	¥667,923	15.15	13.09	¥757,090	13.63	11.19	-¥89,167	1.52	1.90	Dominant
10% discount on drug costs	¥619,399	15.15	13.09	¥705,718	13.63	11.19	-¥86,319	1.52	1.90	Dominant
20% discount on drug costs	¥575,549	15.15	13.09	¥654,346	13.63	11.19	-¥78,797	1.52	1.90	Dominant
30% discount on drug costs	¥531,698	15.15	13.09	¥602,973	13.63	11.19	-¥71,275	1.52	1.90	Dominant

Abbreviations: DMR, Deep Molecular Response; ELN, European Leukemia Net; LY, life year; QALY, quality-adjusted life year.

### Sensitivity analysis results

**Fig 2:**
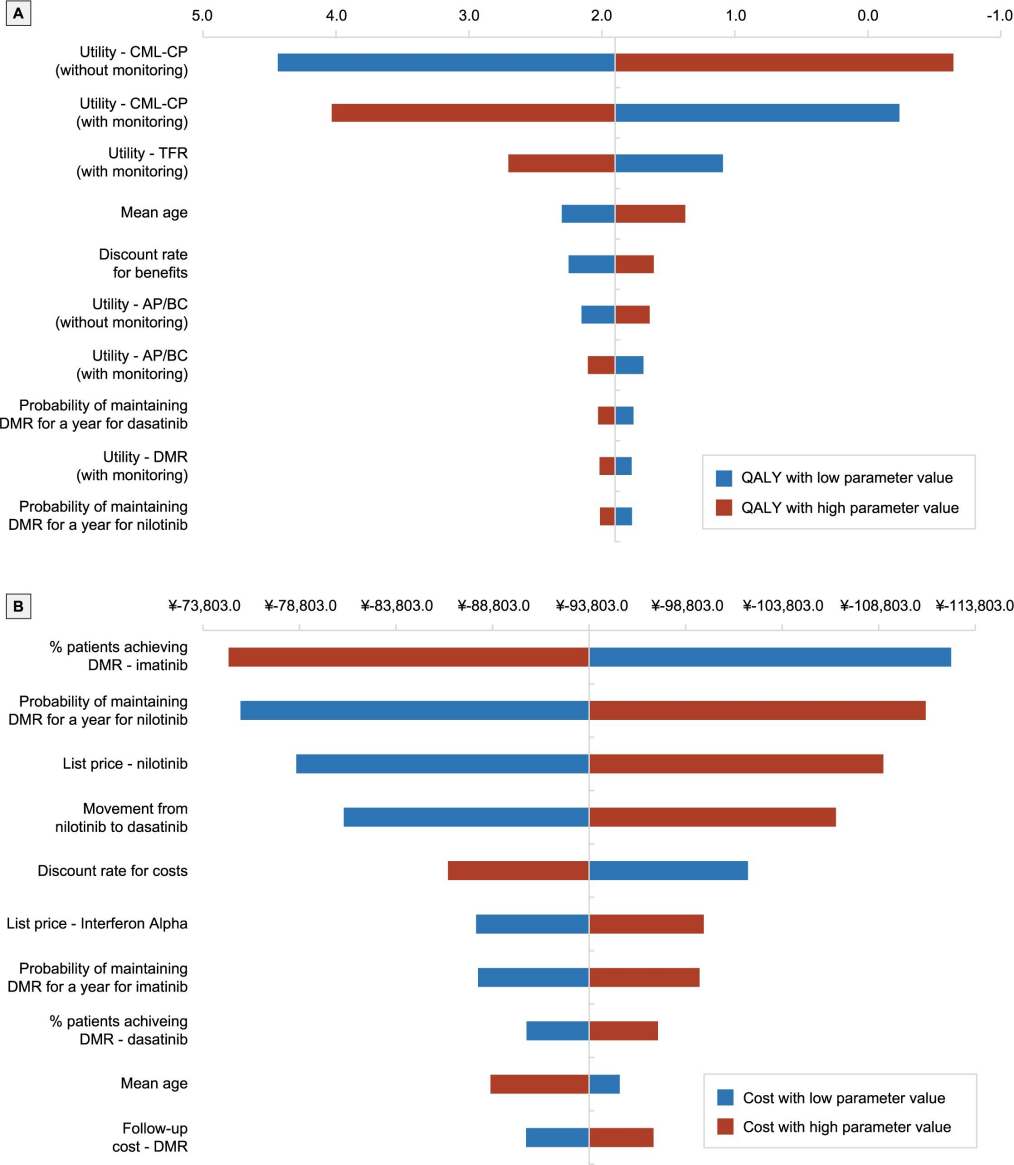
Results of one-way sensitivity analyses. Abbreviations: AP, Accelerated Phase; BC, Blast Crisis; CML-CP, Chronic Myeloid Leukemia; DMR, Deep Molecular Response; QALY, quality-adjusted life year; TFR, Treatment Free Remission.

Sensitivity analysis showed that for QALYs, the model was most sensitive to changes in utility values for CML-CP (Fig 2A). Regarding costs, the model was most sensitive to the proportion of patients achieving DMR with imatinib, the probability of maintaining DMR for 1 year after nilotinib, the list price for nilotinib, and movement from nilotinib to dasatinib (Fig 2A).

## Discussion

The introduction of TKIs has led to a shift in the management of CML, and is associated with a marked improvement in patient survival [9]. CML is now managed as a chronic condition and patients may remain on therapy for the remainder of their life. An increase in the prevalence of CML patients coupled with the long duration of TKI treatment has placed substantial financial burden to healthcare systems. Patients with CML-CP on TKIs who achieve a stable sustained DMR are candidates for discontinuing treatment when accompanied by regular molecular monitoring. Not all countries that provide reimbursement for TKIs, however, also provide reimbursement for molecular monitoring. Presently, the impact of molecular monitoring on the economic burden of CML-CP is unknown. Therefore, this study sought to determine the cost effectiveness of molecular monitoring in patients with CML-CP being treated with standard treatment in China compared to no molecular monitoring from a Chinese payer perspective. Results from our analysis showed that molecular monitoring led to increased LYs and QALYs, and reduced costs over a patients’ lifetime compared to no molecular monitoring. Results were consistent across multiple scenario analyses, including variations in the proportion of patients who achieved DMR and maintained DMR for 1 year. The model was also robust to most parameters including: (i) changes in the utility values for CML-CP with and without monitoring and TFR with molecular monitoring for QALYs, (ii) the proportion of patients achieving DMR with imatinib, (iii) the probability of maintaining DMR for 1 year after nilotinib, (iv) the list price for nilotinib and (v) the movement from nilotinib to dasatinib for costs.

Molecular monitoring has been associated with improved clinical outcomes in several studies. A large, retrospective chart review of patients with CML-CP receiving first-line imatinib therapy (N = 402) reported that regular molecular monitoring resulted in a statistically significantly lower risk of progression and improved PFS compared to no molecular monitoring [28]. Similarly, in a retrospective cohort study of 245 patients with CML-CP who were treated with TKIs, molecular monitoring led to a significantly reduced rate of disease progression or mortality [26]. Importantly, this significant reduction in rate occurred regardless of the level of adherence to treatment. In contrast, other studies note the importance of medical monitoring on treatment adherence, where high levels of adherence to treatment has been associated with good clinical outcomes [59]. In China, a retrospective study that investigated the impact of molecular monitoring frequency and medical insurance coverage on clinical outcomes among patients with CML (N = 335) in the Jiangsu province reported that more frequent molecular monitoring (ie, ≥3 per year) significantly improved the odds of achieving a major molecular response compared to less frequent monitoring (ie, <2 times per year) over 24 months [60].

Evidence from several studies has shown that molecular monitoring is associated with overall cost savings despite the additional monitoring related costs. A study of 901 Japanese patients eligible for TFR after first- or second-line TKI reported a total cost savings of ¥2,577,451,775, ¥2,589,441,684 and ¥2,458,281,181 during years 1, 2 and 3 (total of ¥7,625,174,640 or US$66,567,775) with 100% compliance to molecular monitoring [34]. These cost savings persisted with reduced willingness to try TFR. In a large, retrospective, US claims database study of 1,205 patients with CML-CP, molecular monitoring significantly lowered costs of all-cause and progression-related inpatient admissions and medical service costs compared to no molecular monitoring, after adjustment [61]. Lower costs were attributed to fewer inpatient admissions in patients with regular molecular monitoring than in those with no molecular monitoring. Cost savings have also been associated with patients entering TFR and discontinuing TKI therapy [32,49].

In this study, achieving DMR and the probability of maintaining DMR for 1 year represented substantial drivers in the savings associated with monitoring. In addition to the added cost of molecular monitoring, increased costs were observed for the DMR health state in patients with molecular monitoring. These additional costs were offset by cost savings in the CML-CP health state, as well as cost savings in the AP/BC health state, in terminal care costs, and in follow-up costs. Furthermore, reductions in the proportion of patients who achieved DMR or in the probability of maintaining DMR for 1 year did not impact results, with molecular monitoring continuing to have improved clinical benefits and lower costs compared to no molecular monitoring. Results were consistent even when these parameters were reduced by 30%. Additionally, when the frequency of molecular monitoring was increased to align with the ELN guidelines, overall cost effectiveness remained the same.

The sources of data used to inform the model represent a significant strength of this study. Characteristics of the hypothetical cohort were informed by Chinese registry studies, monitoring frequencies were based on input from key opinion leaders, utilities were informed by previously-published cost-effectiveness analyses, cost inputs were derived from Chinese medical service databanks and health states and transition probabilities were based on published clinical trials. The resource utilization for follow-up costs and terminal care costs used in this study, however, were not based on data from the Chinese population, but rather from the National Institute for Health and Care Excellence and from published literature. Despite accumulating evidence demonstrating that molecular monitoring is associated with increased benefit, our model also conservatively assumed that molecular monitoring did not impact the TTD or PFS curves used to drive transition between health states. The assumptions that patients receiving molecular monitoring at different frequencies incurred similar benefits and the linear treatment algorithm in which flumatinib/dasatinib was considered for first-, second-, or third-line treatment represent the primary limitations of this study. In China, for instance, treatment guidelines recommend flumatinib as a first-line therapy. As such, certain treatment pathways like flumatinib (first line) to nilotinib (second line) were not included. The assumption that all patients eligible for treatment discontinuation agreed to enter TFR also represents a potential limitation of this study. Lastly, the AE costs associated with exposure to TKIs were not considered in this analysis. Given that monitoring leads to treatment discontinuation and reduced risk of AEs, this limitation may also have contributed to an underestimation of the benefits and cost savings incurred by monitoring.

## Conclusion

Overall, this analysis demonstrates that adherence to guideline recommendations for regular molecular monitoring of patients with CML-CP treated with TKIs provides significant clinical benefit that leads to substantial cost savings during the lifetime of a patient compared to no molecular monitoring from the perspective of a Chinese payer. The availability of the low-cost TFR health state to patients that received molecular monitoring was the overarching driver of this result. In a time where healthcare systems have limited resources to allocate for optimal patient care, investment in molecular monitoring is an ideal choice for improving patient benefits at a reduced cost and should go hand in hand with investment in TKIs.

## Supporting information

S1 Fig TTD, PFS and OS curves used to inform the model. (TIF)

S1 Table Clinical inputs for scenario analyses. (TIF)
