# Supplementary figures and images for: Enabling access to molecular monitoring for chronic myeloid leukemia patients is cost effective in China
Source: PLoS One. 2021 Oct 25;16(10):e0259076. doi: 10.1371/journal.pone.0259076 (PMC8544861; doi:10.1371/journal.pone.0259076)

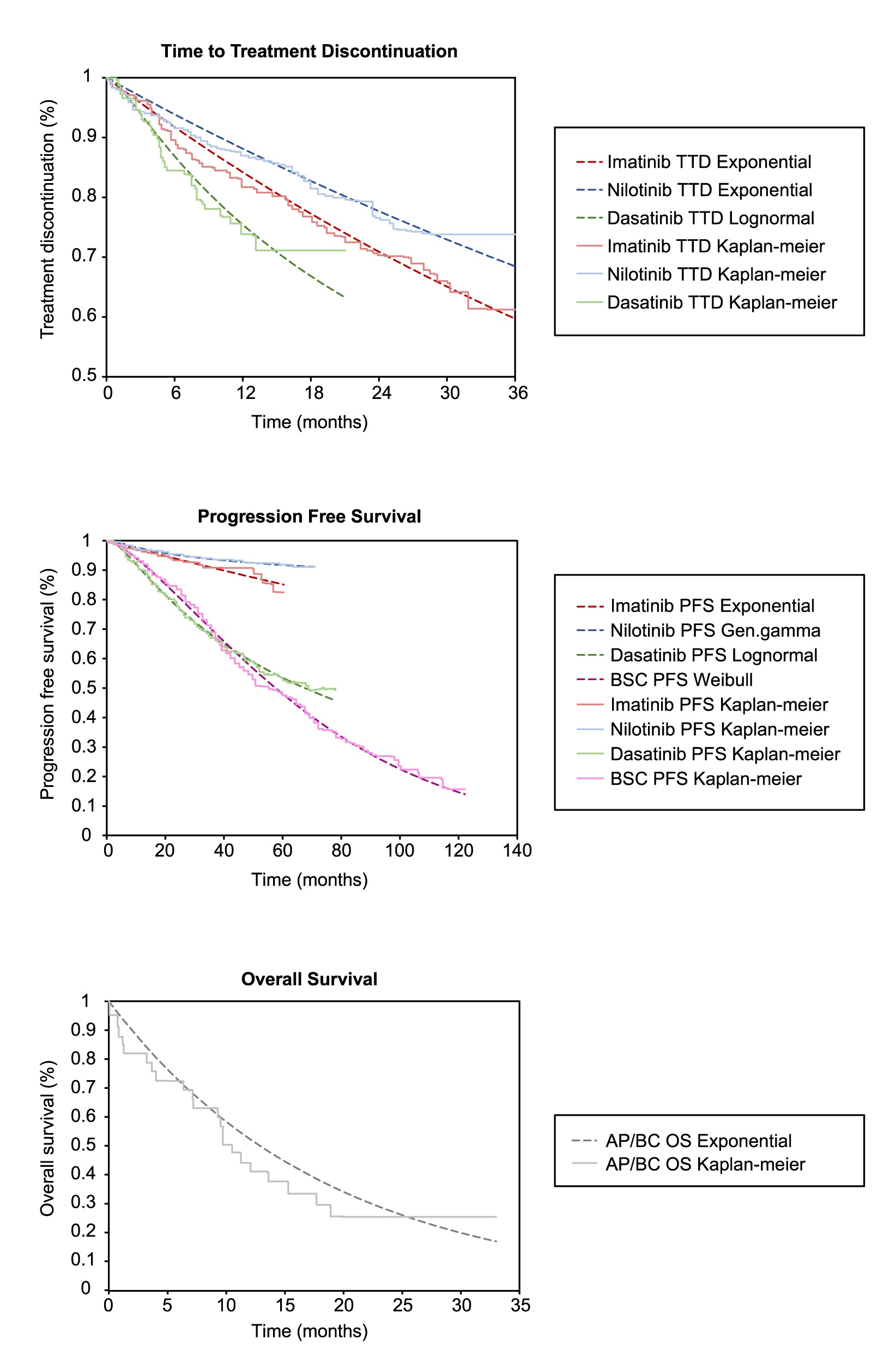

Supplement: S1 Fig — (TIF) [file pone.0259076.s001.tif]

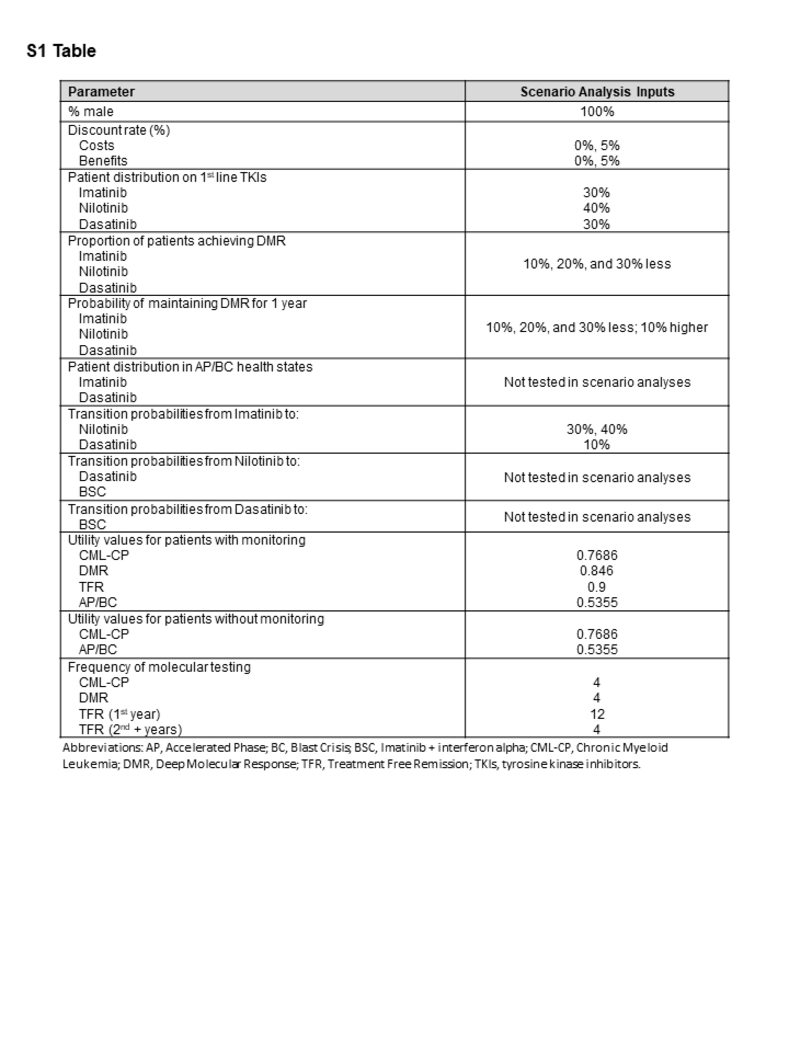

Supplement: S1 Table — (TIF) [file pone.0259076.s002.tif]
